# Epigenetics and cancer disparities: when nature might be nurture

**DOI:** 10.18632/oncoscience.555

**Published:** 2022-04-22

**Authors:** I. King Jordan, Kara K. Lee, John F. McDonald, Leonardo Mariño-Ramírez

**Affiliations:** ^1^

**Keywords:** health disparities, cancer, epigenetics, genomics, race

Despite a sharp overall decrease in cancer mortality, survival disparities between racial and ethnic groups stubbornly persist [1]. Black patients have the highest all-cancer mortality rate in the US, including common breast, colon, lung, and prostate cancer types, even though Whites show the highest overall rate of new cancer cases. Hispanic, Asian/Pacific Islander, and American Indian/Alaskan Native groups all have lower cancer mortality compared to both Black and White groups.

We recently conducted a large-cohort study of cancer survival disparities in the US, leveraging the National Cancer Institute’s Cancer Genome Atlas (TCGA) to power a pan-cancer analysis of 33 cancers for 9,818 patients [2]. We identified four cancer types with significant cancer survival disparities between racial and ethnic groups – breast invasive carcinoma, head and neck squamous cell carcinoma, kidney renal clear cell carcinoma, and skin cutaneous carcinoma – along with seven cancer-related genes that interact with genetic ancestry to contribute to the observed disparities. Here, we highlight the implications of our results pointing to epigenetic mechanisms, DNA methylation in particular, as a potential link between genetic (nature) and environmental (nurture) contributions to cancer health disparities.

The relative importance of nature versus nurture for health disparities remains a matter of considerable debate. While it is widely recognized that individual-level health outcomes result from genetics, environmental factors, and their interactions, a role for genetics in health differences among groups is far more contentious. The Centers for Disease Control and Prevention (CDC) define health disparities as “preventable differences in the burden of disease, injury, violence, or opportunities to achieve optimal health that are experienced by socially disadvantaged populations”; the federal government’s Healthy People initiative similarly defines health disparities as “a particular type of health difference that is closely linked with social, economic, and/or environmental disadvantage”, and the National Institute on Minority Health and Health Disparities (NIMHD) states that “a health disparity is a health difference that adversely affects disadvantaged populations”. All of these definitions emphasize social and environmental contributions to health disparities while eliding possible genetic or biological causes.

Health disparities defined in this way, where an assumed set of causes is baked into the definition, are considered distinct from more generic health differences, which are defined in a way that is independent of possible causes [3]. This distinction between health disparities and health differences is grounded in concerns about the legacy of scientific racism in the field of genetics and an intention to steer health disparities research efforts towards disadvantaged groups, both of which are certainly valid concerns [4, 5]. Nevertheless, we favor a more agnostic approach to health disparities research, one which allows for joint consideration of both genetic and environmental factors, as well as their interactions, i.e. nature AND nurture as opposed to nature versus nurture. We believe that the holistic approach is more likely to yield unexpected results, and to tell us something truly new about the causes of health disparities, than a tautological approach that assumes causes *a priori*. Indeed, our recent results suggest that nature and nurture can not be so easily disentangled, and what may appear to be nature could actually be nurture.

Our study entailed the integration of computational multi-omics techniques – genomics, transcriptomics, and methylomics – and statistical epidemiology based on survival analysis. We first identified cancer survival disparities between patient racial and ethnic groups, and then we statistically modeled how interactions between genetic ancestry and gene mutation, expression, and methylation may have contributed to the observed disparities. Despite clear differences in patterns of genetic ancestry between patients’ self-identified race and ethnicity, we did not find any significant associations with genetic variation and cancer survival disparities. In other words, genetic differences between groups could not explain the observed disparities. There were, however, clear differences in group-specific patterns of DNA methylation and gene expression that were associated with cancer survival disparities. For example, hypomethylation of the *PAQR6* gene promoter region in African ancestry patients is associated with higher gene expression and a greater risk of breast cancer mortality compared to European ancestry patients. Consistent with our own results, the progesterone receptor encoded by *PAQR6* – Progestin and AdipoQ Receptor Family Member 6 – has been shown to mediate progestin-induced inhibition of apoptosis in breast cancer cells [6].

Our results indicate that changes in gene expression mediated by epigenetic mechanisms have a greater contribution to cancer survival disparities than group-specific genetic variants. This is consistent with a previously proposed role for epigenetics in health disparities [7]. According to the epigenetics-health disparities hypothesis, DNA methylation can be altered based on specific environmental exposures, including pollution and psychosocial stress, which are in turn linked to racial and ethnic differences in both exposure levels and health outcomes (e.g., for cardiovascular disease, premature birth, and cancer). DNA methylation levels have been linked to the molecular etiology of all these disease classes in support of this hypothesis. The epigenetics-health disparities hypothesis blurs the distinction between nature and nurture, with environmental exposures leading to DNA-level changes in patients’ genes and their expression. In this sense, epigenetics can serve as a crucial link between group-specific environmental exposures and genetically influenced pathophysiological states that are implicated in health disparities.

It should be noted that our study of TCGA did not include potentially relevant environmental variables, as environmental exposure data are not widely available for this particular dataset. Thus, dispositive proof of the epigenetics-health disparities hypothesis will require an integrative approach that includes robust data on environmental exposures together with patient multi-omics and health outcome data.
